# Genetic Correction of IL-10RB Deficiency Reconstitutes Anti-Inflammatory Regulation in iPSC-Derived Macrophages

**DOI:** 10.3390/jpm11030221

**Published:** 2021-03-20

**Authors:** Dirk Hoffmann, Johanna Sens, Sebastian Brennig, Daniel Brand, Friederike Philipp, Philippe Vollmer Barbosa, Johannes Kuehle, Doris Steinemann, Daniela Lenz, Theresa Buchegger, Michael Morgan, Christine S. Falk, Christoph Klein, Nico Lachmann, Axel Schambach

**Affiliations:** 1 Institute of Experimental Hematology, Hannover Medical School, Carl-Neuberg-Strasse 1, 30625 Hannover, Germany; hoffmann.dirk@mh-hannover.de (D.H.); sens.johanna@mh-hannover.de (J.S.); s.brennig@googlemail.com (S.B.); daniel.brand@mail.de (D.B.); rike.philipp@gmail.com (F.P.); VollmerBarbosa.Philippe@mh-hannover.de (P.V.B.); johannes.kuehle@uk-koeln.de (J.K.); lenz.daniela@gmx.de (D.L.); buchegger.theresa@mh-hannover.de (T.B.); morgan.michael@mh-hannover.de (M.M.); lachmann.nico@mh-hannover.de (N.L.); 2 REBIRTH Research Center for Translational Regenerative Medicine, Hannover Medical School, 30625 Hannover, Germany; 3 Fraunhofer Institute for Toxicology and Experimental Medicine, 30625 Hannover, Germany; 4 Institute of Cell and Molecular Pathology, Hannover Medical School, 30625 Hannover, Germany; steinemann.doris@mh-hannover.de; 5 Transplant Immunology, Hannover Medical School, Carl-Neuberg-Strasse 1, 30625 Hannover, Germany; Falk.Christine@mh-hannover.de; 6 Department of Pediatrics, Dr. von Hauner Children’s Hospital, University Hospital, Ludwig Maximilian University Munich, 80337 Munich, Germany; Christoph.Klein@med.uni-muenchen.de; 7 Division of Hematology/Oncology, Boston Children’s Hospital, Harvard Medical School, Boston, MA 02115, USA

**Keywords:** very early-onset inflammatory bowel disease, IL-10 signaling, disease modeling, gene therapy, gene editing

## Abstract

Patient material from rare diseases such as very early-onset inflammatory bowel disease (VEO-IBD) is often limited. The use of patient-derived induced pluripotent stem cells (iPSCs) for disease modeling is a promising approach to investigate disease pathomechanisms and therapeutic strategies. We successfully developed VEO-IBD patient-derived iPSC lines harboring a mutation in the IL-10 receptor β-chain (IL-10RB) associated with defective IL-10 signaling. To characterize the disease phenotype, healthy control and VEO-IBD iPSCs were differentiated into macrophages. IL-10 stimulation induced characteristic signal transducer and activator of transcription 3 (STAT3) and suppressor of cytokine signaling 3 (*SOCS3*) downstream signaling and anti-inflammatory regulation of lipopolysaccharide (LPS)-mediated cytokine secretion in healthy control iPSC-derived macrophages. In contrast, IL-10 stimulation of macrophages derived from patient iPSCs did not result in STAT3 phosphorylation and subsequent *SOCS3* expression, recapitulating the phenotype of cells from patients with IL-10RB deficiency. In line with this, LPS-induced cytokine secretion (e.g., IL-6 and tumor necrosis factor-α (TNF-α)) could not be downregulated by exogenous IL-10 stimulation in VEO-IBD iPSC-derived macrophages. Correction of the IL-10RB defect via lentiviral gene therapy or genome editing in the adeno-associated virus integration site 1 (*AAVS1*) safe harbor locus led to reconstitution of the anti-inflammatory response. Corrected cells showed IL-10RB expression, IL-10-inducible phosphorylation of STAT3, and subsequent *SOCS3* expression. Furthermore, LPS-mediated TNF-α secretion could be modulated by IL-10 stimulation in gene-edited VEO-IBD iPSC-derived macrophages. Our established disease models provide the opportunity to identify and validate new curative molecular therapies and to investigate phenotypes and consequences of additional individual IL-10 signaling pathway-dependent VEO-IBD mutations.

## 1. Introduction

Inflammatory bowel disease (IBD) is a chronic inflammatory disorder of the gastrointestinal tract. In addition to disease subtypes such as Crohn’s disease (CD) and ulcerative colitis (UC), which are often based on environmental factors and polygenic dispositions, there are variants in which genetic disorders induce a very early-onset IBD (VEO-IBD) [1,2,3,4,5,6,7]. In the past years, it has been shown that a group of VEO-IBD patients harbor genetic defects in the interleukin-10 receptor (IL-10R), which abrogate the anti-inflammatory effect of interleukin IL-10 and lead to the development of severe bowel inflammation within the first six years of life [2,5,6,7]. The IL-10R consists of two IL-10R specific α-chains (IL-10RA) and two constitutively expressed IL-10 receptor β-chains (IL-10RB). Mutations in both chains have been shown to disrupt IL-10R signaling. An IL-10R with functional IL-10RA and IL-10RB is needed for the IL-10 induced anti-inflammatory response via downstream targets, e.g., signal transducer and activator of transcription 3 (STAT3) and suppressor of cytokine signaling 3 (SOCS3). IL-10 signaling limits proinflammatory cytokine secretion (e.g., TNF-α) and is important for control of innate immune cell responses [2]. As IL-10RB also functions as a receptor subunit of IL-22, IL-26, IL-28A, IL-28B, and IL-29, VEO-IBD patients with a defect in IL-10RB show broader inflammatory symptoms, such as skin folliculitis [2,8,9]. Current immunosuppressive and anti-inflammatory therapies were found to be ineffective for VEO-IBD patients who harbor a genetic defect in *IL-10RA* or *IL-10RB*.

So far, only allogeneic hematopoietic stem cell (HSC) transplantation was shown to be effective and is the only curative treatment to restore the anti-inflammatory function lacking in VEO-IBD patients [2]. The development of alternative treatment options is currently hampered by a limitation of suitable, human-relevant disease models to study this complex disease. Therefore, the reprogramming of VEO-IBD somatic cells from individual patients into induced pluripotent stem cells (iPSCs) is a valuable technology to generate a nearly inexhaustible source of patient-specific cells [10]. The potential to differentiate patient-derived iPSCs into almost any cell type of the body has been exploited to investigate disease-associated defects in a wide variety of tissue cells [11]. These analyses can provide new information about disease pathomechanisms and a possibility to examine novel symptomatic or curative treatments, e.g., gene therapy in a patient-specific system. One approach would be the insertion of the healthy *IL-10RB* gene into patient-derived cells using lentiviral gene therapy or insertion of a functional *IL-10RB* cassette into a safe harbor locus using genome editing technologies. Integration of a therapeutic transgene into a human safe harbor, e.g., adeno-associated virus integration site 1 (AAVS1), could provide stable and safe expression of the transgene cassette in iPSCs and their progeny [12]. Our group previously showed the feasibility of innovative therapeutic approaches, such as lentiviral gene therapy or treatment with a selective Janus kinase 1 inhibitor (filgotinib), in iPSC-derived *IL-10RB*-knock-out macrophages [13]. In the case of the IL-10R deficiency-associated VEO-IBD, the investigation of iPSC-differentiated macrophages is of major importance as the dysfunction of IL-10 signaling in these innate immune cells directly leads to a severe form of colitis [14].

In the present study, we aimed to model VEO-IBD in an individual patient-specific setting and characterized the functional influence of the *IL-10RB* defect in VEO-IBD iPSC-derived macrophages. The generated VEO-IBD macrophage disease models were examined for functional IL-10R downstream target phosphorylation (e.g., STAT3), *SOCS3* expression, and cytokine secretion. In addition, possible therapeutic approaches based on lentiviral gene therapy and safe harbor-engineered gene editing in VEO-IBD iPSCs were investigated and compared with respect to the functional reconstitution of IL-10 signaling.

## 2. Materials and Methods

### 2.1. Plasmids

The lentiviral correction vector was cloned by the insertion of a codon-optimized human *IL-10RB* cDNA as an *Age*I-*Sal*I fragment into the lentiviral vector pRRL.PPT.CBX3.EFS.EGFP.pre, which was tested to perform most suitably in iPSC before [15]. In brief, this is a 3rd generation lentiviral SIN vector with an internal hybrid promoter, consisting of the UCOE-derived CBX3 element and the elongation factor 1a short (EFS) promoter, and a downstream PRE (woodchuck hepatitis virus (WHV) post-transcriptional regulatory element).

The AAVS1-specific TALEN (kindly provided by Toni Cathomen, formerly Hannover Medical School, now University Medical Center Freiburg, Freiburg, Germany) was described before [12,16]. The codon-optimized human *IL-10RB* cDNA was also cloned as an *Age*I-*Sal*I fragment into the AAVS1 donor plasmid Donor_AAVS1.CAG.EGFP.RbGpa under the control of a ubiquitous cytomegalovirus early enhancer element and chicken beta-actin promoter (CAG), and a downstream rabbit beta-globin polyadenylation signal (RbGpa), which was kindly provided by Ulrich Martin (Hannover Medical School, Hannover, Germany) and published before [17]. Cloning details are available on request.

### 2.2. Cell Culture

Human embryonic kidney 293T (ATCC^®^ CRL3216^TM^, LGC Standards, Wesel, Germany) cells were cultured in Dulbecco’s modified Eagle’s medium (DMEM) with 10% heat-inactivated fetal bovine serum (FBS), 100 U/mL penicillin, 100 µg/mL streptomycin, and 1 mM sodium pyruvate (all from PAN Biotech, Aidenbach, Germany). C3H murine embryonic feeder cells (MEF; kindly provided by the MPI for Molecular Biomedicine, Muenster, Germany) were cultured in DMEM low glucose (PAN Biotech, Aidenbach, Germany), 15% heat-inactivated FBS, 2 mM L-glutamine (Biochrom AG, Berlin, Germany), 100 U/mL penicillin, 100 µg/mL streptomycin, and 100 µM β-mercaptoethanol (Sigma Aldrich, Seelze, Germany). Healthy and patient-derived iPSCs were cultured on MEF cells in iPSC medium (DMEM/F12 with 20% knock-out serum replacement (KO-SR) (both Gibco, Karlsruhe, Germany), 1% Non-Essential Amino Acids Solution (NEAA; Invitrogen, Karlsruhe, Germany), 2 mM L-glutamine, 100 U/mL penicillin, 100 µg/mL streptomycin, 100 µM β-mercaptoethanol, and 20 ng/mL human β-fibroblast growth factor (bFGF; kindly provided by Institute for Technical Chemistry, Leibniz University Hannover, Hannover, Germany). Before harvesting the iPSCs, cells were treated for 1 h with 10 µM Y-27632 (Rho-Associated Protein Kinase Inhibitor; Tocris, Bristol, UK). After iPSC splitting, Y-27632 was added for one day to the culture medium. Medium was exchanged every day.

### 2.3. Reprogramming of Patient Fibroblasts and Genotyping

Patient fibroblasts were collected after written informed consent by the Department of Pediatrics of Hannover Medical School, Hannover, Germany. On day 1 of reprogramming, patient-derived human fibroblasts were cultured in fibroblast medium (DMEM with low glucose, 20% FBS, 2 mM L-glutamine, 1% NEAA, 100 U/mL penicillin, 100 µg/mL streptomycin, 100 µM β-mercaptoethanol) with 4 µg/mL protamine sulfate (Sigma Aldrich, Taufkirchen, Germany), 2 mM valproic acid (VPA; Ergenyl; Sanofi-Aventis, Frankfurt, Germany), and 50 µg/mL 2-phospho-L-ascorbic acid (p-vitamin C; Sigma Aldrich, Taufkirchen, Germany) and were spin-inoculated with the reprogramming vector pRRL.PPT.SF.OKSM.idTom.pre FRT [18] (this lentiviral vector harbors an SFFV (spleen focus forming virus promoter)-driven excisable expression cassette with *OCT4*, *KLF4*, *SOX2* and *c-MYC* reprogramming factors) with a multiplicity of infection (MOI) of 1 at 2000 rpm and 37 °C for one hour. On day two, the medium was exchanged to MEF medium with 2 mM VPA and 50 µg/mL p-vitamin C. On day six, cells were cultivated in ½ MEF medium and ½ iPSC medium supplemented with VPA and p-vitamin C. On day eight, cells were transferred to C3H fibroblasts for further cultivation in iPSC medium supplemented with VPA (until day twelve) and p-vitamin C. Medium was exchanged every other day until embryonic stem cell (ESC)-like colonies arose. Several colonies, i.e., iPSC clones, were manually picked and three clones were selected for functional analysis named IBD1, IBD3, and IBD5 iPSCs. The iPSC clone C16 was described before and served as healthy control in several disease studies associated with defects in macrophage functions [19,20,21,22].

To detect the patient-specific mutation in reprogrammed iPSCs, gDNA was isolated using the Blood gDNA extraction kit (Qiagen, Hilden, Germany) and end-point PCR followed by Sanger sequencing was performed to amplify the genomic IL-10RB locus with 5′ CTACCCTTCTTAGCCATGTCA 3′ and 5′ TCCGATCAGATCTTTTGACTC 3′ primers originally used with patient samples [2].

### 2.4. Embryoid Body Formation and Macrophage Differentiation

The detailed protocol for myeloid and macrophage differentiation of iPSC was described before [23]. Briefly, for embryoid body (EB) formation, iPSCs were expanded and harvested with Dispase I (Roche, Darmstadt, Germany). Cells were transferred to Falcon tubes, and cell clumps were resuspended in EB-medium (Knock-out DMEM (Thermo Fisher Scientific, Darmstadt, Germany), 20% KOSR, 2 mM NEAA, 2 mM L-glutamine, 100 U/mL penicillin, 100 µg/mL streptomycin, and 100 µM β-mercaptoethanol supplemented with Y-27632 (10 μM)). Suspension of small iPSC aggregates was transferred to suspension plates. The cells were cultured for three to five days on a Celltron orbital shaker (Infors HT, Einsbach, Germany) at 100 rpm within an incubator at standard culture conditions. After formation, EBs were picked and transferred to adherent tissue culture plates (TPP, Trasadingen, Switzerland). Cells were incubated for one week in EB-myeloid differentiation medium consisting of X-VIVO 10 (Lonza, Basel, Switzerland), 50 µM β-mercaptoethanol, 100 U/mL penicillin, 100 µg/mL streptomycin, 25 ng/mL human interleukin 3 (IL-3), and 50 ng/mL human macrophage colony-stimulating factor (M-CSF) (both PeproTech, Hamburg, Germany). Myeloid suspension cells were produced by a so-called myeloid cell-forming complex (MCFC), harvested as suspension cells, and terminally differentiated to macrophages in Roswell Park Memorial Institute 1640 medium (RPMI 1640; PAN-Biotech, Aidenbach, Germany), 10% FBS 100 U/mL penicillin, 100 µg/mL streptomycin, and 100 ng/mL M-CSF. Myeloid suspension cells could be harvested periodically after the start of the differentiation.

### 2.5. Flow Cytometric and Cytospin Analysis

Cells were stained for surface markers using antibodies detecting human CD11b, CD14 (both eBioscience, San Diego, CA, USA), CD45 (BioLegend, San Diego, CA, USA), and IL-10RB (BD Biosciences, Heidelberg, Germany) by flow cytometry with the BD FACS Calibur (BD Biosciences, Heidelberg, Germany) or for CD163 (Miltenyi Biotec, Bergisch Gladbach, Germany) and CD86 (BioLegend, San Diego, CA, USA) by flow cytometry with the CytoFLEX Flow Cytometer (Beckman Coulter, Krefeld, Germany). Gating was performed on isotype or fluorescence minus one (FMO) controls. Flow cytometric data analysis was performed by using FlowJo software (LLC, Ashland, OR, USA).

Terminally differentiated macrophages were spun onto glass slides, air-dried, and stained in May–Grünwald and Giemsa staining solution (both Sigma-Aldrich, Taufkirchen, Germany) according to the manufacturer’s protocols. Pictures were taken with a BX51 microscope equipped with an XC50 camera and proceeded with the software Cell^F version 3.4 (all Olympus, Hamburg, Germany).

### 2.6. RT qPCR: SOCS3, OCT4, NANOG, and DNMT3B Expression

Total RNA of iPSC and stimulated iPSC-derived macrophages were isolated by using the RNAzol^®^ protocol (Sigma Aldrich, Taufkirchen, Germany). Isolated RNA was reverse transcribed with the QuantiTect Reverse Transcription Kit according to manufacturer’s instructions (Qiagen, Hilden, Germany). Gene expression was quantified by QuantiTect SYBR Green RT-PCR Kit (Qiagen, Hilden, Germany) using the StepOnePlus Real-Time PCR System (Applied Biosystems, Darmstadt, Germany). We used the following primers for detection of *OCT4* (5′-CCTCACTTCACTGCACTGTA-3′ and 5′-CAGGTTTTCTTTCCCTAGCT-3′), *NANOG* (5′-TCACACGGAGACTGTCTCTC-3′ and 5′-GAACACAGTTCTGGTCTTCTG-3′), *DNMT3B* (5′-ATAAGTCGAAGGTGCGTCGT-3′ and 5′-GGCAACATCTGAAGCCATTT-3′) expression [24]. Expression of *SOCS3* was analyzed in macrophages stimulated with IL-10 (20 ng/mL; Peprotech, Hamburg, Germany) for 2 h using the following primers: 5′ GGAGACTTCGATTCGGGACC 3′, and 5′ GAAACTTGCTGTTGTGGGTGACC 3′ [25]. Gene expression was evaluated as ΔΔCt relative to expression of a housekeeping gene β-*ACTIN/ACTB* (Primer: 5’ CCTCCCTGGAGAAGAGCTA 3′ and 5′ TCCATGCCCAGGAAGGAAG 3′) [26].

### 2.7. Teratoma Formation Assay

For the teratoma formation assay and the subsequent injection of IBD1, IBD3, or IBD5 iPSCs into mice, cells were incubated for two hours with Y-27632 (10 μM) before harvesting. Harvested iPSCs were resuspended in an injection medium with 20 μM Y-27632 and mixed with Corning^®^ Matrigel^®^ Basement Membrane Matrix (Corning, Corning, NY, USA). 3 × 10^6^ cells were injected subcutaneously in both flanks. Mice were sacrificed after teratomas reached around 1.5 cm in diameter. Teratomas were dissected and fixed in 4% paraformaldehyde. Teratomas were embedded in paraffin and cut into 10 μm slices, and hematoxylin-eosin staining was performed with hematoxylin solution modified according to Gill III (Merck, Darmstadt, Germany). Images were made with BX51 microscope, camera XC50, and software Cell^F version 3.4 (all Olympus, Hamburg, Germany).

### 2.8. Quantification of Cytokine Secretion by Bio-Plex Assay and TNF-α ELISA

Macrophages were cultured on adherent plates for Bio-Plex assay (5 × 10^4^ cells/96-well) and analyzed for cytokine secretion 2, 6, and 24 h after LPS (100 ng/mL), or LPS (100 ng/mL) and IL-10 (100 ng/mL) treatment. Supernatants were collected for analyses and stored at −20 °C until use. Supernatants were analyzed for the presence of 27 different proteins with the Bio-Plex Pro™ human Cytokine 27-plex Assay Kit (Bio-Rad, Hercules, CA, USA) according to the manufacturer’s protocol.

For TNF-α secretion analyses, macrophages (5 × 10^4^ cells/96-well) were stimulated with LPS, or LPS and IL-10 for 6 h. Supernatants of stimulated macrophages were analyzed for TNF-α proteins with an enzyme-linked immunosorbent assay (ELISA) Kit (Invitrogen). The analysis was performed according to the manufacturer’s protocol with SpectraMax 340PC (Molecular Devices, San José, CA, USA).

### 2.9. Western Blot Analysis STAT3/pSTAT3

Western blot analyses were performed to detect pSTAT3, STAT3, and α-Tubulin expression in iPSC-derived macrophages after IL-10 (20 ng/mL) stimulation. Thirty minutes after stimulation, cells were washed and detached. Cell pellets were resuspended in lysis buffer (50 mM HEPES, 150 mM NaCl, 50 mM NaF, 10 mM Na_4_P_2_O_7_, 10% glycerin, 1% Triton X-100) with 1 µL of Halt™ Protease Inhibitor Cocktail (Thermo Fisher Scientific). Protein lysate was loaded on a polyacrylamide gel, and gel electrophoresis was performed. Samples were blotted for 1.5 h at 4 °C (400 mA) onto nitrocellulose membranes. Membranes were blocked for 1 h at RT with 5% bovine serum albumin (BSA; PAA, Pasching, Austria) for pSTAT3 or 3% milk powder in PBS (Carl Roth, Karlsruhe, Germany) for STAT3 and α-Tubulin staining. STAT3 (1:2000, Cell Signaling Technology, Frankfurt, Germany), pSTAT3 (1:1000, Cell Signaling Technology, Frankfurt am Main, Germany), or α-Tubulin (1:10,000, Abcam, Cambridge, UK) antibody staining was performed overnight at 4 °C. Secondary antibody staining (Goat-anti-mouse 1:5000 or goat-anti-rabbit 1:5000; Abcam, Cambridge, UK) was performed according to the manufacturer’s description for 1 h at room temperature. Proteins were detected with the SuperSignal West Pico Chemoluminescent Substrate (Thermo Fisher Scientific, Darmstadt, Germany) with a FusionFX instrument (Peqlab, Darmstadt, Germany).

### 2.10. Lentiviral Vector Production

For lentiviral vector production, transfection of 293T cells was performed by using the calcium phosphate method in the presence of 15 mM HEPES (PAA) and 25 μM chloroquine (Sigma-Aldrich). Lentiviral vector plasmids were transfected with expression plasmids for human immunodeficiency virus (HIV) group-specific antigens and viral enzymes (Gag/Pol; pcDNA3.GP.4xCTE), regulator of expression of virion proteins (Rev; RSV-Rev; kindly provided by Thomas Hope, Chicago, IL, USA), and the envelope protein vesicular stomatitis virus (VSVg) (all helper plasmids produced by Plasmid Factory, Bielefeld, Germany). Briefly, 5 × 10^6^ 293T cells were transfected with 5 μg of the LV vector, 12 μg of Gag/Pol, 5 μg of Rev, and 1.5 μg of VSV-G-encoding plasmids; 36–48 h after transfection, supernatants containing lentiviral particles were harvested and filtered through Millex-GP 0.22 μm filters (Millipore, Darmstadt, Germany).

### 2.11. Lentiviral Vector Correction of Patient-Derived iPSC

For LV correction, VEO-IBD iPSC clone 3 (IBD3) was transduced with the lentiviral correction vector pRRL.PPT.CBX3.EFS.IL10RBco.pre using an MOI 3. In more detail, iPSCs were treated with Y-27632 one hour before single-cell harvesting using 0.05% trypsin and 0.02% EDTA. Cells were transduced in iPSC medium containing Y-27632 and 4 mg/mL protamine sulfate for 1 h and thereby kept in suspension by gently flicking every 10–15 min. Afterwards, cells were transferred to C3H feeder cells and grown in iPSC medium. Medium was supplemented with Y-27632 for the first day. After a sufficient number of iPSC colonies were grown, cells were sorted by FACS in the Research Facility Cell Sorting at Hannover Medical School, Hannover, Germany. Transduced iPSCs were positively stained by the IL-10RB antibody. Transgene expressing iPSCs were subjected to two rounds of cell sorting before macrophage differentiation experiments and functional analyses.

### 2.12. Targeting and Genotyping of the AAVS1 Locus for Correction of VEO-IBD iPSC

For targeted integration of the IL-10RB transgene cassette into the AAVS1 locus of VEO-IBD iPSC, the clone IBD3 was nucleofected using a formerly described protocol [12]. Briefly, 2 × 10^6^ iPSCs were nucleofected with 2.5 µg of pAAVS1-specific TALEN plasmid (kindly provided by Toni Cathomen, formerly Hannover Medical School, now University Medical Center Freiburg, Freiburg, Germany) [12] and 2.5 µg of the donor plasmid Donor_AAVS1.CAG.IL10Rco.RbGpa using the Amaxa Nucleofector II and the mouse ES Cell Nucleofection Kit according to the manufacturer’s protocol (both Lonza, Cologne, Germany). Nucleofected iPSCs were expanded, and single cells were sorted by FACS for constitutive transgene expression using the IL-10RB antibody staining. Validation of the AAVS1-targeted integration was performed with isolated gDNA from iPSCs using the Blood gDNA extraction kit (Qiagen). End-point PCR analysis was performed with a primer set to detect the AAVS1 locus 5′ and 3′ targeted integration, random plasmid integrations, and the AAVS1 unmodified (“wild type”) locus. Primers were described by our group in detail before [12].

### 2.13. Statistical Analysis

Statistical analyses were performed using GraphPad Prism software (GSL Biotech, Chicago, IL, USA). Two-way ANOVA and unpaired *t* test were used for statistical comparison. *p*-values > 0.05 were considered not significant (ns). *p*-values ≤ 0.05 (*) were considered significant, *p*-values ≤ 0.01 (**) were considered very significant, and *p*-values ≤ 0.001 (***) were considered extremely significant.

## 3. Results

### 3.1. Generation of iPSC from IL-10RB-Deficient VEO-IBD Patient Cells

**Figure 1:**
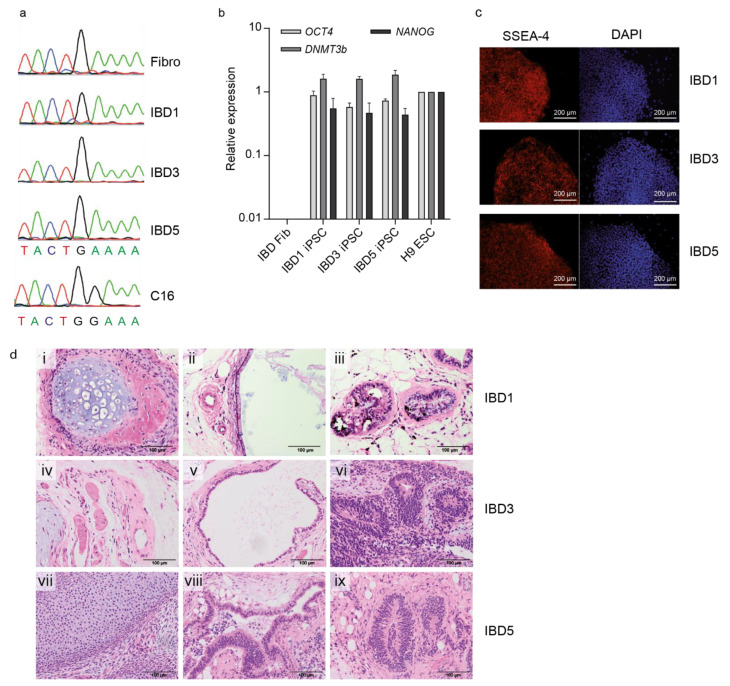
Generation of iPSCs from an *IL-10RB*-/- VEO-IBD patient. (**a**) Sanger sequencing to detect the IL-10RB mutation (c.G477A) in patient’s fibroblasts (Fib) and VEO-IBD iPSC clones (IBD1, IBD3, IBD5). Healthy C16 iPSC served as a control. (**b**) RT-qPCR expression analysis of endogenous pluripotency genes *OCT4*, *NANOG*, and *DNMT3b* in VEO-IBD iPSC and fibroblasts in relation to H9 ESCs (set to one, mean of technical replicates). (**c**) Immunofluorescence microscopy to detect pluripotency marker SSEA-4 expression on the surface of IBD iPSCs. DAPI was used for nuclei staining. Bars correspond to 200 µm. (**d**) Microscopic analyses of teratoma tissue generated from VEO-IBD iPSC clones after subcutaneous injection into immunodeficient mice. Hematoxylin and Eosin staining indicated mature tissues derived from all three germ layers: IBD1 (i)–(iii): cartilage (mesoderm, (i)), intestinal epithelium (endoderm, (ii)), neural tissue (ectoderm, (iii)). IBD3 (iv)–(vi): smooth muscle (mesoderm/ectoderm, (iv), ciliated airway epithelium (endoderm, (v)), neural tissue (ectoderm, (vi)). IBD5 (vii)–(ix): cartilage (mesoderm, (vii)), intestinal epithelium (endoderm, (viii)), neural tissue (ectoderm, (ix)). Bars correspond to 100 µm.

VEO-IBD iPSC models were generated by reprogramming male patient fibroblasts that harbored an IL-10RB defect. The generated and selected VEO-IBD iPSC clones (IBD1, IBD3, and IBD5) were analyzed by Sanger sequencing to confirm the patient-specific mutation in *IL-10RB* (c.G477A, p.Trp159X). All three generated iPSC clones showed the congenital mutation apparent in the parental fibroblasts (Figure 1a). To verify their iPSC status, the expression of endogenous pluripotency genes octamer-binding transcription factor 4 (*OCT4*), *NANOG*, and DNA methyltransferase 3 beta (*DNMT3b*) were analyzed and compared to the expression in human fibroblasts as negative and H9 ESCs as positive controls. The generated IBD1, IBD3, and IBD5 iPSC clones showed an expression level of the pluripotency-specific genes comparable to the H9 ESC line. Patient-derived VEO-IBD fibroblasts showed no expression of the analyzed genes (Figure 1b). Furthermore, the patient-specific clones (IBD1, IBD3, and IBD5) were stained with the early pluripotency marker stage-specific embryonic antigen-4 (SSEA-4) to further verify the embryonic stem cell-like character of the generated cells. Analysis by fluorescence microscopy confirmed that the cells showed a strong expression of SSEA-4 (Figure 1c). Teratoma formation, which is characteristic of pluripotent cells differentiating into cells of all three germ layers, was demonstrated for all three VEO-IBD patient-derived clones after subcutaneous injection into immunodeficient mice. The injected cells were able to form tissues derived from all three germ layers (endoderm, ectoderm, and mesoderm) (Figure 1d). In conclusion, these various analyses clearly demonstrate that the generated VEO-IBD patient-derived cell lines IBD1, IBD3, and IBD5 are fully reprogrammed iPSC. Importantly, the three generated clones did not exhibit aberrations on the genomic level, as demonstrated by array CGH (Figure S1).

### 3.2. Differentiation of VEO-IBD Patient-Derived iPSCs (IL-10RB-/-) into Macrophages

**Figure 2:**
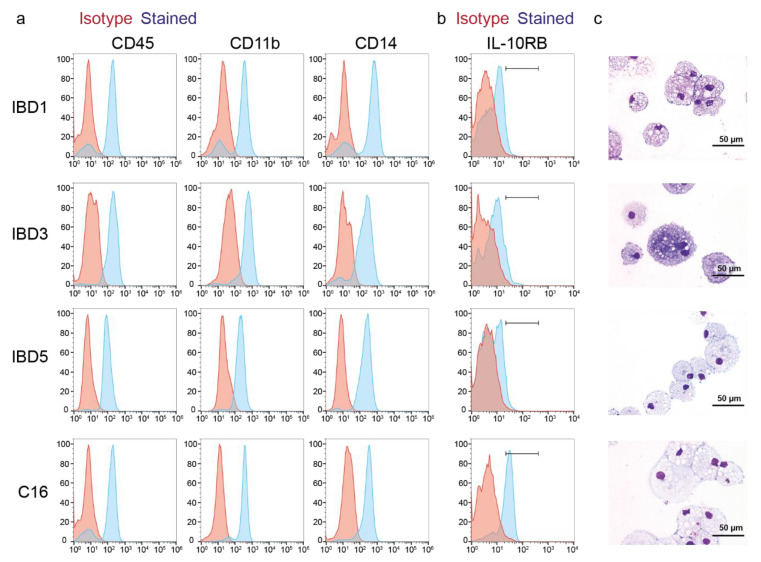
Macrophage differentiation of *IL-10RB*-/- VEO-IBD iPSCs. (**a**) Detection of macrophage surface marker expression (CD45, CD11b, CD14) on differentiated hematopoietic cells derived from healthy control (C16) and VEO-IBD (IBD1, IBD3, IBD5) iPSCs (red line, isotype; blue line, surface marker). (**b**) IL-10RB surface expression on iPSC-derived monocytes/macrophages analyzed by flow cytometry (red line, isotype; blue line, surface marker). (**c**) Morphological analyses by Pappenheim staining and light microscopy (bars correspond to 50 µm).

As defects in the IL-10R and IL-10 signaling on macrophages play a major role in causing severe colitis in patients, the generated VEO-IBD patient-derived iPSC clones were differentiated into macrophages for the detailed analysis of the IL-10RB defect. Therefore, IBD1, IBD3, and IBD5 iPSCs were differentiated by EB formation and MCFC according to previously published protocols (see Material and Methods). Flow cytometric analysis of the iPSC-derived cells showed that mature macrophages (CD45^+^/CD11b^+^/CD14^+^) were obtained after differentiation from VEO-IBD IPSC clones (Figure 2a). Additionally, VEO-IBD patient-derived macrophages (IBD1, IBD3, and IBD5) showed abrogated IL-10RB expression on their surface in comparison to macrophages derived from the healthy C16 control, which had clearly detectable surface expression of IL-10RB (Figure 2b). Pappenheim staining of cytospins revealed that VEO-IBD-patient-iPSC-derived cells had typical macrophage morphology (e.g., vacuolated cytoplasm) (Figure 2c), which further showed efficient generation of iPSC-derived macrophages.

### 3.3. Dysregulated Immune Response in VEO-IBD iPSC-Derived Macrophages

**Figure 3:**
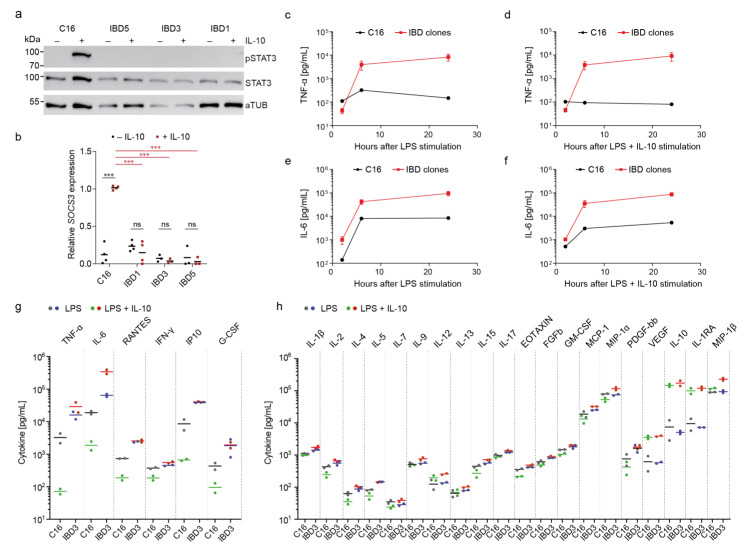
Deficient immune regulation in VEO-IBD iPSC-derived macrophages. (**a**) Detection of STAT3 phosphorylation (pY705; pSTAT3) upon LPS stimulation in iPSC-derived macrophages. Expression of STAT3 and alpha-Tubulin (aTUB) as controls. (**b**) Detection of IL-10-induced *SOCS3* mRNA expression in healthy and IBD-iPSC-derived macrophages by qRT-PCR (mean of biological replicates, *n* = 3; *** *p* ≤ 0.001 two way-ANOVA and Holm–Šídák multiple comparison test). (**c**) Screening of TNF-α secretion by LPS-stimulated iPSC-derived macrophages over time using Bio-Plex assay (mean ± SD of IBD clones; *n* = 3). (**d**) Secretion of TNF-α upon LPS and IL-10 co-stimulation of macrophages over time (mean ± SD of IBD clones; *n* = 3). (**e**,**f**) Detection of IL-6 secretion by LPS, or LPS and IL-10 stimulated iPSC-derived macrophages (mean ± SD of IBD clones; *n* = 3). (**g**) Bio-Plex-based detection of IL-10 regulated inflammatory cytokines 6 h after LPS, or LPS and IL-10 co-stimulation of iPSC derived macrophages (biological replicates, *n* = 2). (**h**) Moderate or no effect of IL-10 on LPS-stimulated cytokine secretion as detected by Bio-Plex analysis (biological replicates, *n* = 2).

Defects in *IL-10RB* are described to cause severe colitis, as several anti-inflammatory processes are regulated by IL-10 binding to the IL-10R and subsequent STAT3 phosphorylation and activation. To characterize STAT3 functionality in the VEO-IBD iPSC model, macrophages were stimulated with IL-10. Subsequently, STAT3 phosphorylation and total STAT3 expression were examined by Western blot. IBD1, IBD3, and IBD5 macrophages showed STAT3 expression, but no STAT3 phosphorylation upon IL-10 stimulation. In contrast, STAT3 was efficiently phosphorylated in healthy C16 macrophages after IL-10 stimulation (Figure 3a). As phosphorylation of STAT3 leads to the activation of anti-inflammatory SOCS3 signaling, which in turn inhibits, for example, proinflammatory TNF-α, IL-10-induced *SOCS3* mRNA expression in healthy control and VEO-IBD iPSC-derived macrophages was measured by qRT-PCR. Healthy C16 macrophages showed strong *SOCS3* expression after IL-10 stimulation, whereas IBD1, IBD3, and IBD5 macrophages lacked any induction of *SOCS3* expression (Figure 3b). Due to the lack of IL-10RB, the VEO-IBD iPSC-derived macrophages showed deficiencies within the IL-10R/STAT3/SOCS3 pathway.

To determine the duration of LPS stimulation necessary to generate proinflammatory responses in the patient iPSC VEO-IBD model, we analyzed the secretion of TNF-α and IL-6 after LPS stimulation either with or without concomitant IL-10 stimulation. Healthy and VEO-IBD iPSC-derived macrophages were stimulated with LPS for 2, 6, and 24 h. In the healthy C16 macrophages, the highest LPS-induced TNF-α secretion was detected after 6 h of stimulation and started to decrease after 24 h (Figure 3c). In contrast, no increase in TNF-α secretion was observed at any time point following simultaneous stimulation of healthy macrophages with LPS and IL-10 (Figure 3d). In comparison to the healthy C16 control cells, VEO-IBD patient-derived iPSC models showed much higher TNF-α secretion levels, which increased sharply after 6 h and continued to moderately increase up to 24-h (Figure 3c). In contrast to the healthy C16 control cells, simultaneous LPS and IL-10 stimulation did not result in reduced TNF-α secretion from IBD macrophages at any time point measured (Figure 3d). As another proinflammatory marker, we investigated IL-6 and found that IL-6 secretion reached a maximum after 6 h of LPS stimulation in healthy iPSC-derived macrophages (Figure 3e). Further, LPS stimulation resulted in higher IL-6 secretion in macrophages from VEO-IBD patient clones compared to healthy C16 iPSC-derived macrophages, which could not be decreased by IL-10 stimulation (Figure 3e,f). To further characterize the impaired immunoregulatory phenotype of the generated VEO-IBD patient models, secretion of a broader panel of cytokines by healthy C16 and IBD3 macrophages were analyzed 6 h after LPS or LPS and IL-10 stimulation. In healthy C16 macrophages, LPS-induced secretion of TNF-α, IL-6, CCL5/RANTES, IFN-γ, CXCL10/IP-10, and G-CSF were highly decreased after IL-10 stimulation, which was in stark contrast to VEO-IBD patient-derived macrophages (Figure 3g). Moreover, LPS induced the secretion of many more immunomodulatory cytokines (IL-1β, IL-2, IL-4, IL-5, IL-7, IL-9, IL-10, IL-12, IL-13, IL-15, IL-17, CCL11/EOTAXIN, FGFb, GM-CSF, CCL2/MCP-1, CCL3/MIP-1α, CCL4/MIP-1β, PDGF-bb, VEGF, IL-10, and IL-1RA) in healthy and VEO-IBD-derived macrophages. A slight reduction of LPS-induced IL-2, IL-4, IL-5, IL-7, IL-15, EOTAXIN, GM-CSF, CCL2/MCP-1, CCL3/MIP-1α, and PDGF-bb secretion by additional IL-10 stimulation was detected in healthy C16-macrophages, but not in IBD3-macrophages. IL-10 had no apparent effect on IL-1β, IL-9, IL-12, IL-13, IL-17, and FGFb secretion from healthy or VEO-IBD macrophages. Interestingly, increased secretion of IL-12, VEGF, IL-1RA, and MIP-1β was observed after IL-10 stimulation of healthy and VEO-IBD macrophages (Figure 3h).

### 3.4. Genetic Correction of VEO-IBD iPSC Restored Functional Anti-Inflammatory Responses in iPSC-Derived Macrophages

**Figure 4:**
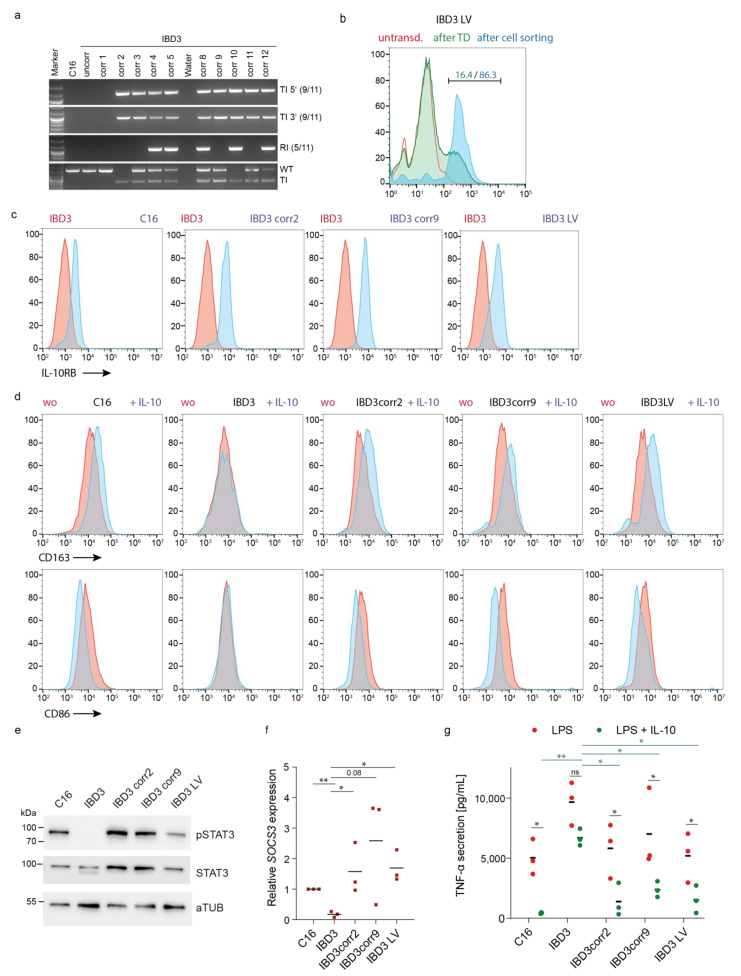
Genetic correction of iPSCs resulted in functional anti-inflammatory recovery of iPSC-derived macrophages. (**a**) Genotyping of the donor cassette integration into the AAVS1 genomic locus. PCR analysis using oligonucleotides to detect the right and left flanking arms of the donor cassette in IBD3 subclones to investigate 5′ and 3′ targeted integration (TI). Unspecific random integration (RI) and detection of the physiological wild type (WT) or targeted AAVS1 (TI) locus were used for more detailed validation of the loci. (**b**) Flow cytometric analysis of IL-10RB surface expression on lentiviral vector transduced IBD3 iPSC (IBD3 LV) before and after purification of transgene expressing cells (red line, untransduced iPSCs; green line, iPSCs after transduction (TD); blue line, sorted iPSCs by FACS). (**c**) Flow cytometric analysis of IL-10RB expression on corrected and uncorrected myeloid-differentiated suspension cells (red line, IBD3-derived cells; blue line, genetic corrected iPSC-derived cells). (**d**) IL-10 specific CD163 and CD86 up- or downregulation on uncorrected or corrected iPSC-derived macrophages upon stimulation (red line, without stimulation; blue line, stimulation with IL-10). (**e**) Western blot analysis of pSTAT3 phosphorylation (pY705, pSTAT3) in iPSC-derived macrophages after IL-10 stimulation. Expression of STAT3 and alpha-Tubulin (aTUB) as controls. (**f**) Upregulation of *SOCS3* mRNA expression upon IL-10 stimulation in iPSC-derived macrophages detected by qRT-PCR (mean of biological replicates, *n* = 3; * *p* ≤ 0.05, ** *p* ≤ 0.01 unpaired *t*-test). (**g**) IL-10-mediated regulation of TNF-α secretion in LPS-stimulated uncorrected and corrected iPSC-derived macrophages (mean of biological replicates, *n* = 3; * *p* ≤ 0.05, ** *p* ≤ 0.01 two-way ANOVA and Holm–Šídák multiple comparison test).

Possible therapeutic gene addition approaches based on gene editing (by targeting the safe harbor AAVS1 locus) and lentiviral vector-based gene therapy were investigated and compared according to their efficiency to reconstitute IL-10RB expression and IL-10 signaling. The VEO-iPSC clone IBD3 was nucleofected with an AAVS1 locus-specific TALEN, as previously described by our group for the correction of another genetic defect in iPSCs, and a donor plasmid with a human codon-optimized cDNA for IL-10RB. Expression of the therapeutic gene was mediated by a ubiquitous cytomegalovirus early enhancer element and chicken beta-actin (CAG) promoter (Figure S2a). Transfected and IL-10RB-expressing iPSCs were single cell-sorted by FACS, expanded, and analyzed for the targeted integration (TI) of the transgene cassette (Figure 4a). As a further quality control of gene-edited iPSCs, PCR analyses to detect random integration (RI) of the donor plasmid were performed. To detect clones with monoallelic or biallelic integrations of the transgene cassette, the targeted (TI) and (untargeted) wild type (WT) loci were analyzed in the single clones. In summary, nine of eleven clones exhibited a targeted integration of the cassette. However, five of these clones also contained a random integration of the donor plasmid, and were thus excluded from further analyses. Two clones that were free of random integration were chosen for further analysis, i.e., IBD3corr2 (corr for corrected) with a biallelic and IBD3corr9 with a monoallelic integration of the cassette.

For lentiviral gene therapy, the iPSC IBD3 clone was transduced with a codon-optimized *IL*-*10RB* transgene cassette driven by the elongation factor 1α short (EFS) promoter, which was juxtaposed to a minimal ubiquitous chromatin opening element (CBX3-UCOE; short: CBX3) (Figure S2b). Of note, this vector architecture was previously tested to perform best in iPSCs to prevent epigenetic vector silencing, and this promoter combination was demonstrated to be robustly expressed and less silenced during differentiation. Transduced iPSCs were sorted for IL-10RB expression mediated by the constitutive CBX3-EFS promoter of the lentiviral correction vector (Figure 4b). Positively gene-edited and vector-transduced iPSCs were then used for differentiation into macrophages.

IL-10RB expression in LV-corrected and TALEN-mediated AAVS1 gene-edited iPSC-derived myeloid suspension cells was detected by flow cytometric analysis. Both correction mechanisms led to successful reconstitution of IL-10RB expression in myeloid suspension cells (Figure 4c). However, AAVS1 gene-edited myeloid cells exhibited slightly higher expression of the receptor compared to healthy and LV-corrected cells. Functionally, we detected CD163 upregulation and CD86 downregulation on healthy and corrected iPSC-derived macrophages upon IL-10 stimulation, which was not the case on uncorrected VEO-IBD macrophages (Figure 4d). To verify whether the IL-10 signaling pathway was restored in IL-10RB-reconstituted cells, the activation and/or expression of the downstream targets STAT3 and *SOCS3* were analyzed in corrected and uncorrected iPSC-derived macrophages. IL-10 stimulation of the VEO-IBD disease models showed restored STAT3 phosphorylation (Figure 4e) and subsequent *SOCS3* expression (Figure 4f) in LV-corrected and AAVS1 gene-edited (IBD3 corr2, IBD3 corr9) VEO-IBD disease models. Furthermore, the restored *SOCS3* expression levels were partially higher in corrected VEO-IBD models than in healthy C16 macrophages (Figure 4f). Notably, LPS-mediated TNF-α secretion could be regulated upon IL-10 stimulation in corrected VEO-IBD models (Figure 4g). No major differences in TNF-α downregulation were observed between the biallelic-edited IBD3corr2 and the monoallelic-edited IBD3corr9 clone or the LV-corrected iPSC-derived macrophages. However, the downregulation in healthy macrophages was slightly more pronounced.

## 4. Discussion

VEO-IBD is a primary immunodeficiency that affects children early after birth and results in severe chronic inflammation of the gut. Several VEO-IBD patients exhibit a defect in the IL-10 signaling pathway [2,3,5,6,7,14]. Macrophages play a major role in the control of inflammatory responses, and their inflammatory dysregulation leads to the onset of infant inflammatory bowel disease [14,27]. Hyperinflammatory responses accompanied by high levels of cytokine secretion especially affect the gut in these very young patients. This is due to a non-functional IL-10 signaling pathway, which impairs the mucosal immune system and gut homeostasis [27]. The interaction of host immunity and intestinal microbiota—the mechanisms that regulate tolerance of indigenous microbiota and elimination of pathogenic bacteria—does not function properly in these patients. The IL-10 pathway and the consequences of dysfunctional IL-10 receptor signaling were intensively studied in mouse models, which provided conclusive insights into principal functions [14,27]. However, differences between human and rodent IL-10-mediated mucosal immune tolerance or the phenotype of individual patient-specific mutations cannot be studied in these murine models, which necessitates the development of human models. However, these inherited defects are very rare, and thus the patient-derived material needed for research to develop a better understanding of disease mechanisms as well as the development of new therapeutics is scarce.

In this study, we established a meaningful human disease model for VEO-IBD in a patient-specific context to investigate an individual human VEO-IBD mutation in detail and to apply this model to validate new therapeutic options. By reprogramming fibroblasts from a young child with a genetic defect in *IL*-*10RB* (c.G477A, p.Trp159X), we generated an inexhaustible source of three different iPSC clones, which can be differentiated to produce VEO-IBD macrophages (Figure 1 and Figure 2). The impaired inflammatory regulation and dysregulated cytokine secretion in these patient-specific cell models were very pronounced. In our study, we showed that LPS from Gram-negative bacteria stimulated secretion of the proinflammatory cytokines TNF-α and IL-6 by macrophages with a different dynamic over time (Figure 3). Proinflammatory cytokine secretion levels were highly enhanced in the VEO-IBD model compared to the healthy control. Importantly, the secretion of these inflammatory cytokines was not downregulated by IL-10 and remained high over time, while the secretion of the inflammatory cytokines was effectively downregulated by IL-10 in healthy control cells. The dysfunction of the IL-10 signaling pathway was also shown in the VEO-IBD model, which is important as IL-10 defects induced the first steps of the anti-inflammatory response. Our disease model exhibited a strong phenotype on the level of STAT3 phosphorylation and induction of *SOCS3* mRNA expression, both of which are critical downstream effectors of IL-10 receptor signaling. Our iPSC-derived macrophage model provided additional information about the LPS-induced secretion of inflammatory cytokines such as TNF-α, IL-6, RANTES, IP-10, and G-CSF. Importantly, while secretion of inflammatory cytokines was strongly downregulated in healthy control macrophages by IL-10, downregulation was impaired in the VEO-IBD context. Of note, our study also showed that IL-10 caused only moderate or no effects on the regulation of other typical proinflammatory cytokines (Figure 3h). This phenotype strongly replicates findings in primary blood samples of the patient from whom these cells were derived [2]. In this aspect, the strong phenotype in our VEO-IBD disease model provided a meaningful read-out system to test new therapeutic applications to treat this severe disease.

We applied our system to test and validate two strategies for gene therapy. In one approach, we applied lentiviral gene therapy for the genetic correction of iPSC. As lentiviral vectors are highly affected by epigenetic silencing of the internal promoter in iPSCs and, especially, during iPSC differentiation, the vector cargo and especially promoter elements must be carefully chosen. In former studies, we and others tested innovative hybrid promoters to overcome these restrictions [15,19,28]. The promoter and vector configuration used in our VEO-IBD disease model was validated to be most suitable in the former comparative study [15]. Using this vector, we observed a high level of reconstituted IL-10RB expression in macrophages after hematopoietic differentiation of vector-transduced VEO-IBD iPSCs (Figure 4b). The strategy to generate stable long-term cultures of lentiviral vector expressing iPSCs was different from our former studies, in which we transduced hematopoietic progenitors derived from CRISPR-Cas9-mediated *IL*-*10RB* knock-out iPSCs during the differentiation process with a lentiviral vector driven by SFFV promoter [13]. The latter validation of lentiviral gene therapy in iPSC-derived cells required high numbers of hematopoietic progenitor cells for transduction, and subsequent cell sorting, which necessitated utilization of a transcription factor-mediated hematopoietic forward programming protocol to generate sufficient amounts of cells.

The second gene therapy approach in the current study employed the targeting of the AAVS1 locus, which was formerly described to fulfill safe harbor criteria and to provide the possibility of stable transgene expression in iPSC [12]. However, our earlier work showed that also in this locus, certain promoters are susceptible to transgene silencing in iPSC-derived myeloid cells [29]. We applied the CAG promoter, which was previously described to mediate high expression in the AAVS-1 locus in iPSC and their progeny [17]. Stable IL-10 receptor reconstitution was accomplished using this strategy in our VEO-IBD model. The suitability of AAVS1-targeting has also been demonstrated recently in another study that applied a patient VEO-IBD iPSC model [30]. These cells harbored a different mutation within the *IL-10RB* gene, which led to the absence of the receptor. The study also showed the strength of an iPSC disease model to study disease mechanisms and macrophage dysfunction, e.g., in bacterial killing. In our study, we provide further evidence of the AAVS1-targeting strategy as a therapeutic intervention and additionally investigated safety criteria in addition to the functional benefits of gene correction. Clearly, the selection of transgene expressing iPSC clones after AAVS1 targeting can be accompanied by random integration of the donor plasmid. These unwanted genetic modifications have been precisely analyzed in several clones to rule out unwanted side effects, e.g., genotoxicity by promoter-mediated activation of neighboring genes. Furthermore, we focused on the functional analysis of mono- and biallelic integration of the therapeutic cassette to address dose questions. No major differences were observed between one or two integrations of the transgene, indicating that a single transgene cassette, driven by the CAG promoter, is sufficient to reconstitute IL-10RB receptor expression (Figure 4c). IL-10RB expression was even enhanced on gene-edited compared to healthy control cells. These differences did not affect IL-10-mediated CD163 up- or CD86 downregulation on iPSC-derived macrophages; however, effects of IL-10RB expression strength on macrophage polarization have to be further elucidated. Comparison of lentiviral gene therapy and AAVS1 genome targeting showed that both strategies led to functional reconstitution of the inflammatory regulation in patient-derived macrophages, including induction of STAT3 phosphorylation, *SOCS3* mRNA expression, and, importantly, downregulation of LPS-induced TNF-α secretion upon IL-10 stimulation (Figure 4e,f). However, in genetically corrected VEO-IBD macrophages, the ability to downregulate TNF-α was slightly less pronounced compared to healthy control macrophages. Further optimization of donor and vector architecture using alternative promoters or regulatory elements could improve the efficiency of anti-inflammatory fine-tuning and recovery. Generally, genome targeting, which enables controlled and site-specific integration, is expected to be more precise than lentiviral gene therapy, which harbors the risk of insertional mutagenesis caused by the general random vector integration in the genome. However, designer nucleases also harbor a risk of genotoxicity, e.g., mediated by unwanted random integration and off-target effects. In recent years, CRISPR-Cas9 opened new avenues for genome editing for basic research and clinical applications [31,32]. The Cas9 nuclease is directed to the genomic DNA target via site-specific synthetic single-guide RNAs, which are easy to design. Therefore, this technology provides the possibility for genome-specific correction of single mutations related to gene defects. Our VEO-IBD is most suitable to demonstrate and validate the potential of CRISPR-Cas9 mediated genome editing and to compare its efficiency to the lentiviral gene therapy and safe harbor-engineered gene editing. Gene therapy provides the possibility of curative treatment of primary immunodeficiencies as demonstrated for, e.g., severe combined immunodeficiency [33] or Wiskott–Aldrich syndrome [34]. The suitability of gene therapy for VEO-IBD is indicated by our iPSC disease model, and our results are likely translatable for the applicability of future cell and gene therapies. Furthermore, new treatment possibilities are of high demand as VEO-IBD children suffer tremendously from severe acute inflammation, and common drugs and biologicals have had limited activity in this setting [3,5,6]. Our system provides the possibility to test the applicability of new drugs in a patient-specific setting. Furthermore, our group developed iPSC knock-out models that resembled a VEO-IBD phenotype and studied the effects of small molecules to downregulate the hyperinflammatory phenotype of the system [13].

In summary, iPSC-derived macrophages are highly suitable for studying complex primary immunodeficiencies. This was convincingly demonstrated for pulmonary alveolar proteinosis [20] or mendelian susceptibility to mycobacterial disease [21]. However, in VEO-IBD, not only macrophages are affected by the impaired IL-10 signaling pathway. Investigation of impaired T cell function, especially of regulatory T cells, would be of major importance. Additionally, the barrier function of epithelial cells is impaired in certain IBD patients. These functions were so far studied in an iPSC-derived intestinal epithelial cell model in an immune cell-free environment [35]. Furthermore, Caspase 8 deficiency in three unrelated patients was associated with IBD caused by impaired control of inflammation and intestinal barrier integrity [36]. Therefore, iPSC-based organoid disease models will allow investigation of the orchestrated interactions of epithelial and immune cell functions in the VEO-IBD context.

In conclusion, we established a promising iPSC-based patient-derived VEO-IBD model that resembles the patient phenotype on the level of macrophage dysfunction. This model will provide the opportunity for deeper studies to gain new insight into the complex pathomechanism of this severe disease and to perform proof-of-concept studies for innovative therapies.

## Data Availability

The datasets used and analyzed during the current study are available from the corresponding author upon reasonable request.

## Supplementary Materials

The following are available online at https://www.mdpi.com/2075-4426/11/3/221/s1, Figure S1. Characterization of genomic integrity in VEO-IBD iPSCs; Figure S2. Design of the AAVS1-targeting donor and lentiviral vector.
