# Supplementary material for: Genetic Correction of IL-10RB Deficiency Reconstitutes Anti-Inflammatory Regulation in iPSC-Derived Macrophages
Source: J Pers Med. 2021 Mar 20;11(3):221. doi: 10.3390/jpm11030221 (PMC8003874; doi:10.3390/jpm11030221)

Supplementary Materials:

Genetic correction of IL-10RB-deficiency reconstitutes anti-inflammatory regulation in iPSC-derived macrophages

Dirk Hoffmann [1,2], Johanna Sens [1,2], Sebastian Brennig [1,2], Daniel Brand [1,2], Friederike Philipp [1,2], Philippe Vollmer Barbosa [1,2,3], Johannes Kühle [1,2], Doris Steinemann [4], Daniela Lenz [1,2], Theresa Buchegger [1,2], Michael Morgan [1,2], Christine S. Falk [5], Christoph Klein [6], Nico Lachmann [1,2], Axel Schambach [1,2,7*]

1 Institute of Experimental Hematology, Hannover Medical School, Carl-Neuberg-Strasse 1, 30625 Hannover, Germany
2 REBIRTH Research Center for Translational Regenerative Medicine, Hannover Medical School, 30625 Hannover, Germany
3 Fraunhofer Institute for Toxicology and Experimental Medicine, 30625 Hannover, Germany
4 Institute of Cell and Molecular Pathology, Hannover Medical School, 30625 Hannover, Germany
5 Transplant Immunology, Hannover Medical School, Carl-Neuberg-Strasse 1, 30625 Hannover, Germany
6 Department of Pediatrics, Dr. von Hauner Children's Hospital, University Hospital, Ludwig Maximilian University Munich, 80337 Munich, Germany
7 Division of Hematology/Oncology, Boston Children's Hospital, Harvard Medical School, 02115 Boston, MA, USA
* Correspondence: schambach.axel@mh-hannover.de

SUPPLEMENTARY METHOD

Array-CGH

The genomic integrity of reprogrammed cells was tested by using array CGH (Human Genome Microarray; Agilent Technologies, Waldbronn, Germany). Labeling and hybridization of genomic DNA was performed according to the manufacturer's protocol. Briefly, genomic test DNA was labeled by random priming using the Agilent Genomic DNA Labeling Kit Plus. The test DNA was labeled with Cy3-dUTP and the reference DNA with Cy5-dUTP. Female reference DNA was used for the analysis of IBD1 iPSC (sex mismatched) and male DNA for IBD3 and IBD5 (sex matched). Labeled products were purified by Amicon Ultra 30k filters (Millipore, Billerica, MA, USA), pooled and mixed with human Cot-1 DNA together with 10X Blocking Agent and 2X Hybridization Buffer (Agilent Technologies, Waldbronn, Germany). This solution was hybridized to the microarray probes and washed according to the Agilent protocol. Microarray slides were scanned immediately using an Agilent microarray scanner at a resolution of 2 µm. Fluorescence ratios were calculated using Feature Extraction Software and copy number states analyzed using the CGH data analysis software DNA-Workbench (Agilent Technologies, Waldbronn, Germany).

SUPPLEMENTARY FIGURE LEGENDS

Figure S1. Characterization of genomic integrity in VEO-IBD iPSCs. Array-CGH analysis to detect copy number variation after reprogramming in the iPSC clones (a) IBD1, (b) IBD3, and (c) IBD5. The genomic profiles are shown. x-axis: chromosomes 1-22; X and Y; y-axis: log2 ratios (Cy5/Cy3) representing test/reference.

Figure S2. Design of the AAVS1-targeting donor and lentiviral vector. (a) Schematic illustration of the AAVS1-specific donor cassette for exogenous expression of codon optimized (co) *IL10-RB*. HLA: homology left arm; CAG, cytomegalovirus early enhancer element and chicken beta-actin promoter; pA, polyadenylation signal; HRA, homology right arm. (b) Schematic illustration of the lentiviral vector design. LTR, long terminal repeat; ΔU3, deleted unique 3; R, repeat; U5, unique 5; SD, splice donor; ψ, packaging signal; RRE, Rev response element; SA, splice acceptor; cPPT, central polypurine tract; CBX3, minimal ubiquitous chromatin opening element; EFS, elongation factor 1α short promoter; PRE, post-transcriptional regulatory element.

SUPPLEMENTARY FIGURES

Figure S1

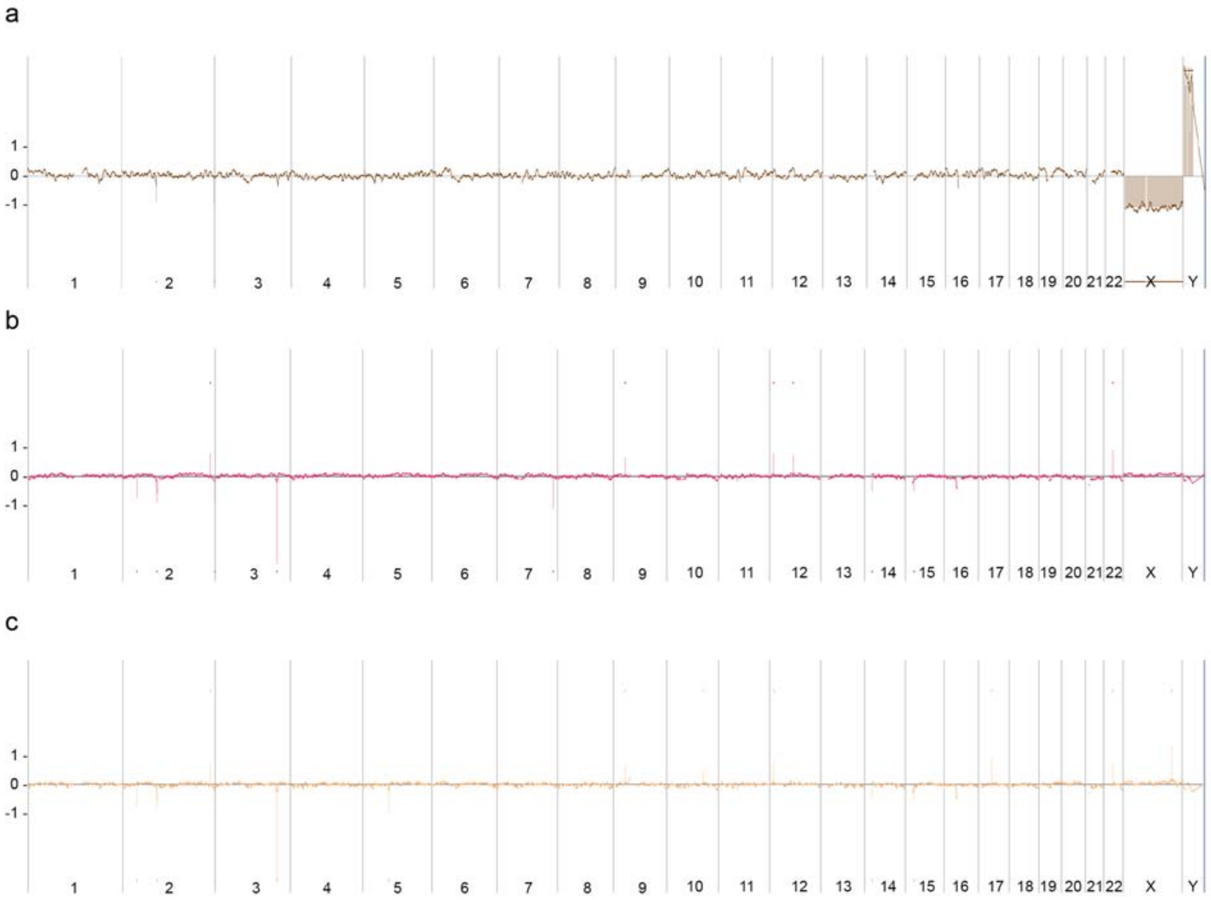

Figure S2

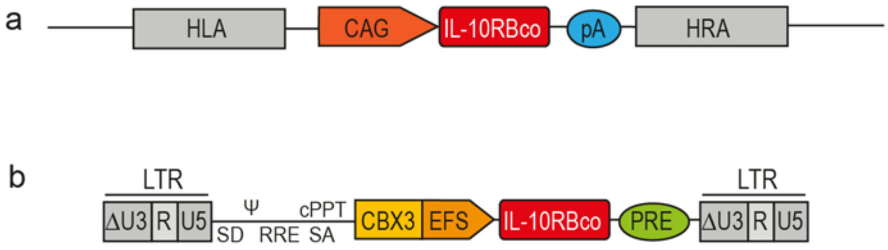

Supplement: Supplementary file 1 [file jpm-11-00221-s001.pdf]
